# Accelerating precision exercise medicine in cancer patients using pooled individual patient data: POLARIS experience

**DOI:** 10.1093/jncics/pkaf078

**Published:** 2025-08-07

**Authors:** Laurien M Buffart, Marlou-Floor Kenkhuis, Robert U Newton, Anne M May, Daniel A Galvão, Kerry S Courneya

**Affiliations:** Department of Medical BioSciences, Radboud University Medical Center, Nijmegen, The Netherlands; Exercise Medicine Research Institute, Edith Cowan University, Joondalup, WA, Australia; Department of Medical BioSciences, Radboud University Medical Center, Nijmegen, The Netherlands; Exercise Medicine Research Institute, Edith Cowan University, Joondalup, WA, Australia; Julius Center for Health Sciences and Primary Care, University Medical Center Utrecht, Utrecht, The Netherlands; Exercise Medicine Research Institute, Edith Cowan University, Joondalup, WA, Australia; Faculty of Kinesiology, Sport, and Recreation, University of Alberta, Edmonton, Canada

## Abstract

Numerous exercise oncology trials have been completed, greatly informing exercise recommendations for patients with cancer. Exercise medicine can be administered in various types, doses, and schedules at various time points. Advancing precision exercise medicine requires understanding of how the effects of different exercise interventions vary by characteristics of individual patients. The Predicting OptimaL cAncer RehabIlitation and Supportive care (POLARIS) study provides an international infrastructure and shared database to perform pooled analyses of individual patient data (IPD) from multiple randomized controlled trials. This commentary aims to highlight the value of pooled IPD analyses, summarize key findings from published pooled IPD analyses on the effects of physical exercise on various outcomes, and provide guidance to advance precision exercise medicine for patients with cancer. POLARIS currently includes IPD from 52 exercise trials. Findings to date indicate that exercise interventions in patients with cancer have beneficial effects on physical fitness, fatigue, health-related quality of life, self-reported cognition (posttreatment), sleep disturbances, and symptoms of anxiety and depression. Additionally, it was determined that the exercise effects varied by characteristics of the patients, including the initial value of the outcome, age, marital status, and education level, and by characteristics of the intervention, including exercise supervision and specificity. Future research opportunities to advance precision exercise medicine for patients with cancer include pooling of trial data from understudied populations, data on clinical outcomes, and biomarkers, as well as applying machine learning models for identifying combinations of covariables that modify intervention effects and predictions of individual treatment effects.

## Background

Over the past decades, cancer incidence has increased substantially. Globally, 1 in 5 people will develop cancer at some point during their life.^1^ In some developed countries, this number has increased to 1 in 2.^2^ Fortunately, survival continues to improve, with 5-year survival rates across all cancers approaching 70% in developed countries,^3^ but large variations by cancer type and stage exist.^1^ Many patients with cancer undergo multiple treatments, which are associated with physical and psychosocial problems, impacting health-related quality of life (HRQoL).^4^

Physical exercise (a subform of physical activity that is planned, structured, and repetitive with the aim to improve physical fitness or health^5^) can effectively mitigate some of the cancer- and treatment-related problems. The landmark randomized controlled trial (RCT) by Winningham and MacVicar in the late 1980s^6-8^ marked the beginning of clinical exercise oncology. Since then, the number of exercise oncology RCTs has increased to 4 in 1996,^9^ 32 in 2005,^10^ 82 in 2010,^11^ and more than 2500 in 2018.^12^ Now the term “exercise oncology” is readily accepted, and in 2024 the International Society for Exercise Oncology was established. Alongside the growth of RCTs, systematic reviews and meta-analyses synthesizing the results have become increasingly central to inform evidence-based clinical practice guidelines.^13^

To improve physical and psychosocial functioning and HRQoL of patients with cancer, leading organizations from multiple countries now recommend that exercise be a part of standard care and provide first guidance on exercise types, doses, and schedules (ie, exercise prescriptions) and precautions to maximize exercise safety.^12^^,^^14-22^ Ideally, exercise prescription should be optimally tailored to the health priorities, characteristics, needs, capabilities, and preferences of individual patients to maximize effectiveness.^23^ Advancing precision exercise oncology requires understanding of how the effects of different exercise prescriptions vary by characteristics of the patient and the phase of the cancer continuum.^24^ Unfortunately, most exercise oncology trials include fewer than 100 patients, making them underpowered to detect interaction (subgroup) effects that are necessary to inform precision exercise oncology.

The Predicting OptimaL cAncer RehabIlitation and Supportive care (POLARIS) initiative, launched more than a decade ago, aimed to address this challenge.^25^ POLARIS provides an international infrastructure and shared database of individual patient data (IPD) from numerous exercise oncology RCTs, enabling investigations of variations in exercise intervention effects across subgroups of patients with cancer. In the absence of large-scale phase 3 trials with adequate power to examine interaction effects, pooled analyses on IPD from multiple RCTs are the preferred method because of larger samples sizes and the ability to separate within and across-trial information and to standardize analytic techniques across the included studies.^26-29^

In this commentary, we aim to highlight the value of pooled IPD analyses, summarize key findings from published pooled IPD analyses on the effects of physical exercise on various outcomes, and provide guidance to advance precision exercise medicine for patients with cancer. To contextualize the pooled IPD analyses, we first describe the history and ongoing management of POLARIS.

## History and management of POLARIS

The POLARIS initiative was launched in 2012. A steering committee and international advisory board were established, and data-sharing policies and procedures were developed.^25^ Literature searches were performed to identify RCTs that examined the exercise effects on HRQoL compared with a usual care, waitlist, or attention control group. Principal investigators of those trials were requested to share IPD of their RCT for pooled analyses. The first search yielded 34 exercise oncology RCTs, of which IPD was pooled after performing data checks and analyzed subsequently. These 34 RCTs were a representative sample of the RCTs that had been conducted so far, and first results were published in 2017.^30^ Original searches also focused on psychosocial interventions, of which IPD of 22 studies were retrieved and analyzed.^31^ After the first series of publications, more exercise oncology RCTs have been included in the POLARIS database, identified via literature searches or personal communication. The 52 RCTs included to date were performed at different phases in the cancer continuum, that is, presurgery, during treatment, posttreatment, and long-term survivorship^32^ (Table S1). Additionally, the database is currently being enriched with IPD of RCTs examining the effects of combined exercise and dietary interventions (unpublished data).

After signing data-sharing agreements, anonymous or pseudonymized IPD from RCTs are securely stored at a digital research environment. Researchers can submit a paper proposal according to the POLARIS policy document that is sent to the consortium, to request permission of using the data for the proposed purpose. Subsequently, researchers can obtain access to the digital research environment to perform the analyses within the environment. Principal investigators of trials that are eligible for the proposed analyses are invited to contribute actively. Key findings of the analyses conducted so far are summarized below.

## Summary of key findings from pooled exercise oncology trial data

The analyses on pooled IPD from trials performed as part of the POLARIS study showed that exercise interventions in patients with cancer have beneficial main effects on physical fitness,^33^ fatigue,^34^ QoL,^30^ physical function,^30^ self-reported cognitive functioning (posttreatment only),^35^ sleep disturbances,^36^ and symptoms of anxiety and depression.^37^ Overall, the average effects on these outcomes were small. However, the effects varied substantially by patient and exercise intervention characteristics, highlighting the need to target exercise to specific patient populations and outcomes. The outcomes and moderators investigated to date are summarized in Table 1, and the key subgroup findings from the pooled IPD analyses are summarized in Table 2. Differences in intervention effects were largely related to exercise supervision and specificity, a patient’s initial value of physical fitness, fatigue and HRQoL, age, marital status, and educational level. These findings provide important guidance to improve precision exercise prescriptions, which are discussed below.

**Table 1.** Overview of the moderators investigated in the POLARIS initiative.

Moderators	Moderator defined by
*Sociodemographic*	
Age	Continuous and categorized
Sex	Categorized into male vs female
Marital status	Categorized into unmarried or living alone vs married or living with partner
Education level	Categorized into low–medium vs high
*Clinical*	
Cancer type	Categorized into breast, male genitourinary, hematological, gastrointestinal, gynecological, respiratory tract, or other types
Type of cancer treatment	Categorized into surgery, chemotherapy, radiotherapy, hormone therapy, or stem cell transplantation; all dichotomized into previous or current treatment vs no treatment
Presence of distant metastases	Categorized into yes vs no
ADT duration^a^	Categorized into acute (<6 months) and chronic ADT exposure (≥6 months)
Gleason score^a^	Categorized into 7 and ≥8
Time since diagnoses^a^	Continuous and categorized into tertiles
*Psychophysiological*	
BMI	Continuous and categorized into underweight (<18.5 kg/m^2^), normal weight (BMI ≥ 18.5 to <25 kg/m^2^), overweight (BMI ≥ 25 to <30 kg/m^2^), obese (BMI ≥30 kg/m^2^)
Low skeletal muscle index^a^	Categorized into ≤7.26 kg.m^−2^, ie, pre-sarcopenia vs ≥7.26 kg.m^−2^
Initial values of the outcome	Continuous and categorized into groups based on clinical cutoff values or tertiles when clinical cutoff values were not available
*Exercise intervention–related*	
Intervention duration	Categorized into ≤12 weeks, >12–24 weeks, and >24 weeks
Intervention timing	Categorized into during treatment and posttreatment
Delivery mode	Categorized into (partly) supervised vs unsupervised
Exercise type	Categorized into AE vs RE vs AE + RE vs RE + impact
Intensity	Categorized into low-moderate and moderate vs moderate-vigorous and vigorous
Frequency	Categorized into <3 vs ≥3 sessions per week (supervised exercise) or <5 vs ≥5 sessions per week (unsupervised exercise)
Duration of exercise session	Categorized into ≤30 min, >30–60 min, or >60 min per session
Volume	Categorized into <150 min per week vs ≥150 min per week
Total no. of sessions^a^	Continuous and categorized based on median value

a Assessed in patients with prostate cancer only.

Abbreviations: ADT = androgen deprivation therapy; BMI = body mass index.

**Table 2.** Summary of patient and exercise intervention characteristics moderating the effects of exercise on various outcomes.

Outcome variable	Key subgroup findings
Aerobic fitness^33^^,^^56^^,^^58^	Exercise has largest effect when it is supervised, includes aerobic exercises, or targets younger people. Exercise *during* treatment has largest effect in patients with better initial values, whereas exercise *following* treatment is beneficial regardless of the initial value.
Lower body muscle strength^33^^,^^56^^,^^58^	Exercise has largest effect when it is supervised, includes resistance exercises, or targets patients without a partner. Exercise *following* treatment has largest effect in patients with worse initial values, whereas exercise *during* treatment is beneficial regardless of the initial value. For supervised exercise, there is a dose-response effect for exercise session duration. *Prostate cancer only:* Resistance-based exercise has larger effects when it targets patients who were younger.
Upper body muscle strength^33^^,^^56^^,^^58^	Exercise has largest effect when it is supervised, includes resistance exercises, or is delivered during treatment. Exercise *following* treatment has largest effect in patients with worse initial values, whereas exercise *during* treatment is beneficial regardless of the initial value. For supervised exercise, there is a dose-response effect for exercise frequency and session duration.
Objectively measured physical function^58^^,^^a^	Resistance-based exercise has larger effect on 400 m walk test and 6 m backwards walk when it targets patients with worse initial values.
Self-reported physical function^30^^,^^56^	Exercise has largest effect when it is supervised or targets patients with worse initial values.
Body composition^54^^,a^	Resistance-based exercise has largest effect on fat mass when it targets patients with worse initial values and who were unmarried.
Blood pressure^54^^,a^	Resistance-based exercise has largest effect on diastolic blood pressure when it targets younger patients.
Lipid profile^54^^,a^	Resistance-based exercise results in largest increases in HDL cholesterol when it targets patients treated with androgen deprivation treatment, prostatectomy, and radiotherapy. Resistance-based exercise results in largest decreases in LDL cholesterol when it targets older patients.
Global quality of life^30^^,^^56^	Exercise has largest effect when it is supervised. Exercise *following* treatment has largest effect in patients with worse initial values, whereas exercise *during* treatment is beneficial regardless of the initial value.
Fatigue^34^^,^^56^	Exercise has largest effect when it is supervised and targets patients with worse initial values.
Self-reported cognitive functioning^35^	Exercise has largest effect when delivered *following* treatment or targets patients without anxiety symptoms.
Sleep disturbances^36^	No significant moderator effects were found.
Anxiety^37^	Exercise has largest effect when it is supervised, includes aerobic exercises, or targets patients with a worse initial value.
Depression^37^	Exercise has largest effect when it is supervised; includes aerobic exercises; and targets patients with a worse initial value, those without a partner, or those with a lower educational level.

a Assessed in patients with prostate cancer only.

### Exercise supervision

The beneficial effects of exercise interventions were consistently larger when they included a supervised component. Supervision by an exercise professional has shown to be important to motivate patients to exercise and to individually tailor the exercise prescriptions to comorbidities and side effects of cancer and its treatment, both contributing to larger adherence.^38-40^ Particularly in a period of intense treatments when patients have compromised physical and mental resilience, lower confidence in their capacity, and have to deal with treatment toxicity, patients particularly benefit from supervision.^38^ Although patient-reported outcomes are prone to placebo effects of increased attention by the exercise professional providing support, studies with attention control groups have still shown supervised exercise effects to be superior.^41-43^

Despite the superior findings of supervised exercise interventions, unsupervised interventions have larger accessibility, particularly in non-metropolitan areas, and are associated with lower costs. Identifying which patient is particularly in need for exercise supervision is important for sustainable implementation. Emerging modalities, such as live-remote supervised exercise (telehealth), provide opportunities to include supervision from an exercise professional in a home-based setting.^44^

It should also be noted that some home-based exercise interventions were designed as behavior change interventions targeting exercise behaviors, whereas supervised exercise interventions were designed to improve physical fitness or health, thereby focusing on different intervention targets and using different approaches. Effective behavior change interventions are usually based on behavior change theories to improve and maintain exercise behavior,^45^^,^^46^ whereas maximizing effects of exercise training interventions relies on the principles of exercise physiology, including exercise specificity, progression, overload, initial values, reversibility, and diminishing returns.^47^ Precision exercise medicine would benefit from incorporating the best of both approaches, that is, paying attention to optimizing exercise training principles while incorporating effective behavior change techniques promoting exercise behavior in the long term.

### Exercise specificity

Findings from the POLARIS study confirmed the importance of the exercise specificity training principle: the exercise effects on aerobic fitness were larger when exercise interventions included an aerobic component and the effects on muscle strength were larger when a resistance exercise component was included. This highlights the importance of targeting the exercise type to the desired outcome. However, in exercise trials among patients with cancer, this principle has not been applied to its full potential.^47^^,^^48^ For example, walking-based interventions have been applied because many patients with cancer have identified walking as the preferred mode of exercise.^23^ Although generally patients adhere better to exercise interventions that meet their preferences, simply walking will not drive positive adaptations in muscle mass and strength for most patients. Likewise, when targeting bone mineral density, exercise prescriptions should include impact loading and resistance exercises targeting specific regions.^49^ Furthermore, the importance of exercise specificity also applies to psychosocial outcomes. For example, findings showed that the exercise effects on anxiety and depression were statistically significant if the program included an aerobic component.^37^ The importance of aerobic exercise in influencing depression has been documented by others^50^ and aligns with current exercise recommendations.^12^

It is important to acknowledge that pooled IPD from trials may have limited power to study differences in effects between different exercise arms because the power thereof generally relies on the number of trials instead of the number of patients due to similar exercise prescriptions within study arms. As the number of trials available for IPD is often lower than the total number of published studies, network meta-analyses is a suitable alternative. By combining direct and indirect evidence within a network of RCTs in 1 analysis, a network meta-analysis facilitates simultaneous comparisons of treatment effects that have not directly been compared head-to-head in a single RCT.^51^^,^^52^ Alternatively, for outcomes that can be assessed in a relatively short time, novel efficient adaptive study designs provide an alternative to traditional study designs to facilitate head-to-head comparisons of different exercise interventions.^53^

### Initial values

Findings from the POLARIS study were that the exercise intervention effects on fatigue, anxiety, depression, and self-reported physical function were larger in patients with worse initial values. This was also the case for global QoL, muscle strength, and self-reported cognitive function when exercise was offered posttreatment. Additionally, resistance-based exercises produced larger decreases in fat mass in patients with prostate cancer with worse initial values.^54^ This aligns with the exercise physiology principle that patients with lower baseline values have more room for improvement (larger window for adaptation) and highlights the importance of targeting exercise interventions to patients most in need and prioritizing the most significant deficits relevant to the patient’s health. However, the analyses also revealed that the role of initial values depends on the timepoint of intervention delivery across the cancer care continuum, as examined with 3-way interaction between intervention, initial value, and timepoint of delivery. Findings were that exercise during treatment prevents deteriorations in global QoL and muscle strength, regardless of the initial value. Thus exercise is also important for people with high initial values, who have more to lose during treatment.^55^ Preventing deteriorations in health, physical fitness, functioning, and HRQoL during treatment has important implications to enhance recovery and return to daily life activities. Additionally, in the metastatic setting, preventing these deteriorations during first-line treatment provides a better starting point and higher resilience to endure second or higher treatment lines.

In contrast to preventing deteriorations by exercise during treatment (ie, intrahabilitation), exercise interventions posttreatment focus on restoring mental and physical abilities, regaining daily functioning and HRQoL (ie, rehabilitation), and promoting health during survivorship including reducing risk of cancer recurrence and development of other chronic diseases (ie, survivorship).^32^ Hence, precision exercise medicine requires consideration of initial values to tailor exercise specifically to outcomes that are relevant to the individual in specific phases within the cancer care continuum.

### Older and unfit patients

Although lower initial values generally provide a larger window for adaptation, findings from POLARIS were that effects of exercise during treatment on aerobic fitness were lower in patients with lower fitness, with no effect in patients who were unfit (ie, [estimated] peak oxygen uptake below 15.4 ml/kg/min).^56^ Effects on aerobic fitness were also smaller in older patients compared with younger patients. From an exercise physiology perspective, improvements in aerobic fitness should be expected in older and unfit patients who perform appropriate type and dose of exercise. Therefore, the lack of effects may be related to lower adherence.^57^ Likewise, analyses in men with prostate cancer revealed that the resistance-based exercise effect on muscle strength was smaller in older patients compared with younger patients, potentially reflective of lower adherence. Additionally, it stresses the importance of a multimodal approach combining exercise with protein intake to improve muscle mass and strength implemented with more sophisticated hypertrophy focused resistance exercise in older people with cancer.^58^

The lack of effects in patients who are unfit is particularly worrisome, because fitness tends to decline during cancer treatment. Hence, this subgroup of unfit patients is at most need of exercise medicine to maintain functional independence, which requires a minimum peak oxygen uptake of 15.4 ml/kg/min for women and 17.7 ml/kg/min for men.^59^ In total, 15% of patients who participated in the exercise oncology trials during treatment analyzed within POLARIS (predominantly performed in women with breast cancer) could be categorized as being below this aerobic fitness threshold. Patients with low fitness were generally older and diagnosed with a more advanced disease.^60^ The poor effectiveness of current exercise programs in unfit or older patients with cancer highlight the need for the development of exercise programs that are better tailored to the needs and capabilities of these patients, for example, by taking into account the multidimensional (physical, mental, social, and cognitive) concepts of frailty.^61^ Multimodal and patient-centered, goal-directed self-management programs were shown to be effective in improving physical function in older people, including those with cancer.^62-64^ As older adults are expected to represent 60% of all new cancer diagnoses in 2035,^65^ personalizing exercise interventions to older populations clearly deserves attention in future research.

### Sociodemographic variations

Next to age, some moderator effects were found for marital status and educational level. Patients without a partner showed larger exercise effects on lower body muscle strength and depressive symptoms compared with patients with a partner. In patients with prostate cancer, a larger effect on fat mass was also found in those without a partner. Explanations for these differences are more likely to be psychosocial than physiological. It may be that patients without a partner benefitted more from the social support provided by the exercise professional delivering the intervention motivating adherence or had to do more weight-bearing activities during daily life activities compared with patients with a partner. Alternatively, other studies have shown larger benefits of exercise in patients with a partner,^66^ which may result from additional motivation and support to attend exercise sessions. Differential effects across marital status highlight the importance of including partners in information provision and intervention delivery.

The effect of exercise on depression varied by education level, with larger effects in patients with lower education. However, education level was not found to moderate other outcomes. This aligns with the finding that cancer prehabilitation was effective across all socioeconomic status (SES) levels.^67^ Nevertheless, education levels and SES are important characteristics to consider for exercise program delivery, as patients with lower levels of education and SES are less likely to participate in programs^68^^,^^69^ or to have proximity and financial access to exercise programs.^70^

## Limitations and future directions of pooled IPD analyses to accelerate precision exercise oncology

Pooled IPD analyses have several advantages and disadvantages that have well been documented.^71^ Over the years, there has been an increased awareness and practices of open science according to the FAIR (Findability, Accessibility, Interoperability, and Reusability) principles,^72^ supporting and facilitating the reuse of IPD from RCTs for pooled analyses. Also, the US Food and Drug Administration and the European Medicines Agency encourage subgroup analyses to explore variability of response to treatment between different patient subgroups within a clinical trial.^73^ This offers ample opportunities to continue this endeavor. Hence, the POLARIS database continues to grow, including exercise RCTs across the cancer care continuum from the moment of diagnosis until death. Researchers can anticipate on global data-sharing by including the required statements in patients’ informed consents.

### Limitations

Some limitations of the pooled IPD analyses using the POLARIS database should be noted. The lower power to examine moderator effects of exercise intervention-related characteristics that are similar across patients within a study (study-level moderators), and the analyses of single moderators have been addressed extensively in our previous publications.^30^^,^^34^ Additionally, evaluations of multiple potential moderators has increased the risk of false-positive or false-negative findings.^73^ Furthermore, as subgroup analyses were not preplanned in each original trial, stratified randomization was not performed. Therefore, we cannot rule out imbalances in moderator variables across study arms. Hence, interpretation requires consideration of biological plausibility, consistency in findings, and clinical relevance of the observed differences.^73^

Moreover, the investigated moderators differed in level of specificity. For example, some sociodemographic or treatment-related variables were rather generic (eg, treatment with chemotherapy or not rather than distinguishing different chemotherapeutic agents). Larger sample sizes per cancer type would allow more specific analyses of sociodemographic, disease-, and treatment-related moderators.^74^ Additionally, due to the lack of information, we were unable to examine moderator effects of potentially relevant variables such as behavioral motivational variables.

Finally, although we started with systematic searches to retrieve trials yielding a representative dataset, we continued with a pragmatic approach, which may have reduced its representativeness.

### Future directions

**Figure 1.**
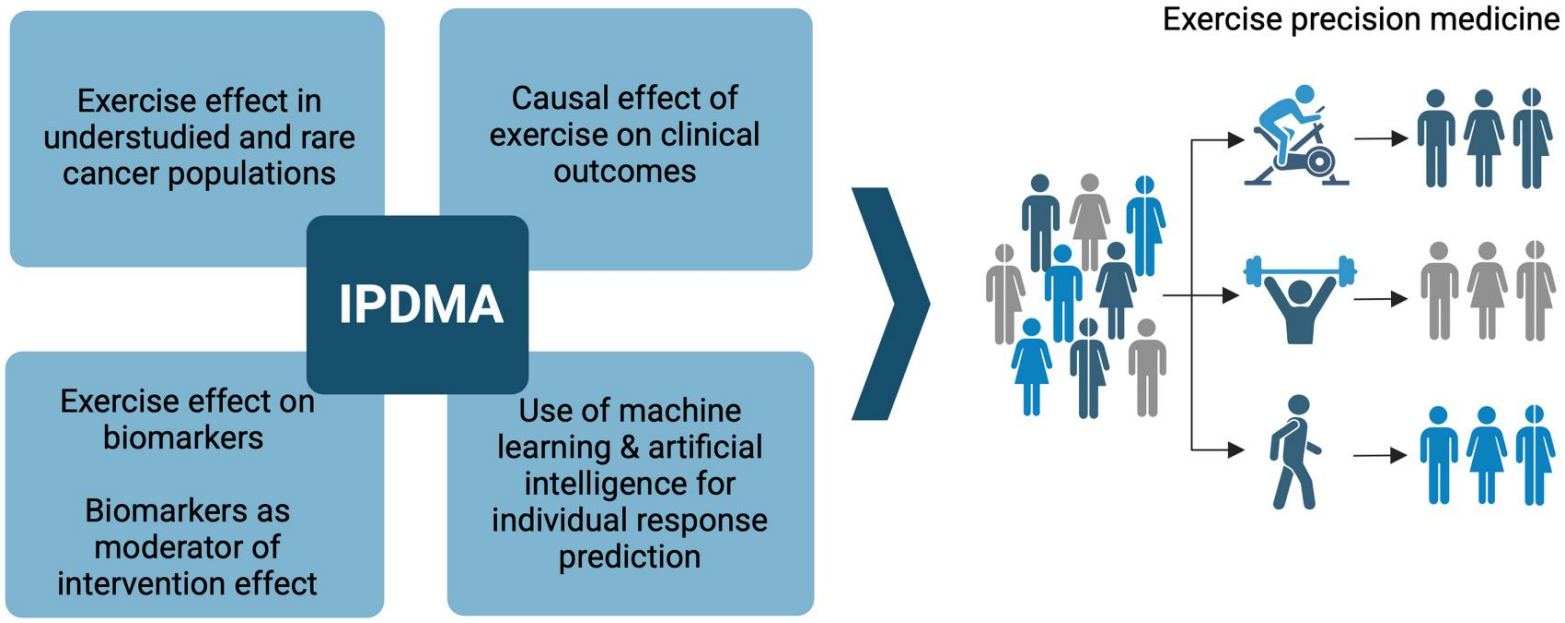
Future directions for pooled individual patient data (IPD) analyses to accelerate exercise precision medicine.

There are multiple opportunities for using pooled IPD to accelerate precision exercise oncology (Figure 1). These include its use for understudied cancer populations, clinical outcomes, and biomarkers. Additionally, with the increasing availability of artificial intelligence (AI) and machine learning (ML) models, the pooled IPD analyses in exercise oncology to date have not been used to its full potential.

#### Understudied populations

Pooled IPD analyses rely on the studies that have been conducted so far, and on investigators willing and able to share IPD, impacting the generalizability of the results. Yet, the potential associated bias is likely larger for estimations of the overall effect on the outcomes than for analyses of the moderators of intervention effects. It should also be noted that most data included in the POLARIS database to date were of patients diagnosed with early-stage breast cancer or prostate cancer, which reflected the studies performed at that point. This highlights the need for exercise oncology RCTs among other cancer patient populations and in patients with metastatic disease, particularly because other disease trajectories and treatment side-effects hamper direct extrapolation of findings.^75^ Fortunately, more studies have been conducted in other cancer patient populations over the past few years. However, they are limited by sample size. Multinational RCTs are good examples to perform RCTs in a reasonable amount of time.^76^ Alternatively, pooling IPD from multiple smaller studies could increase statistical power to examine the effects of exercise on various outcomes in understudied and rare cancer populations. This requires sample size calculations for the separate outcomes to ensure sufficient power.^24^

#### Clinical outcomes

A critical gap in exercise oncology is the lack of knowledge on the causal effects of exercise on treatment outcomes (eg, treatment efficacy, toxicity and toxicity-induced treatment modifications, tumor response) and survival (disease-free, progression-free, overall). Knowledge thereof is pivotal for implementing exercise as integral part of cancer care.^77^ Performing exercise RCTs with clinical endpoints as primary endpoints are difficult in terms of time and resources. For example, the Colon Health and Life-Long Exercise Change (CHALLENGE) trial with disease-free survival as primary endpoint needed 15 years for patient recruitment.^78^ Although such trials are extremely relevant, it is unlikely that they can be conducted in all cancer populations. However, an increasing number of exercise oncology RCTs have included survival outcomes as secondary outcomes,^79-81^ which can be used for pooled IPD analyses.^71^ Likewise, pooled IPD analyses can be applied to increase statistical power to evaluate the effects of exercise on toxicity-induced treatment modifications, which are also more commonly examined as secondary endpoints in exercise oncology trials.^82^ The availability of IPD will also allow further examination of differential impacts of exercise on various types of treatments or chemotherapeutic agents.

#### Biomarkers

Mechanism-based medicine is essential for refining precision exercise medicine, as this helps to identify effective intervention components and to provide causal explanations of how interventions achieve their effects, thereby informing extrapolation of findings to other cancer types. Several preclinical and clinical studies have been performed to identify how exercise can enhance treatment efficacy and impact tumor growth.^83^^,^^84^ These studies have investigated exercise intervention effects on systemic milieu, immune cell function and tumor infiltration, and tumor microvessel density and hypoxia.^85-92^ Unfortunately, human studies were often constrained by small sample sizes due to the complexity and costs of the measurements. Pooled IPD analyses have huge potential to synthesize data across studies examining exercise intervention effects on these biomarkers and linking such alterations to clinical outcomes. Advances in statistical methods will enable mediation analyses on pooled IPD^93^ to understand whether exercise-induced changes in biomarkers and cancer suppression mediate the exercise effects on treatment outcomes and survival.

Additionally, pooling data of biomarkers also enable investigations of their moderating role on various outcomes and identifications of genetic or molecular subgroups of patients who benefit most from specific exercise prescriptions.^94^ For example, observational studies have found differences in the association between physical activity and breast cancer mortality between subgroups of different molecular subtypes.^94^

#### ML models and AI

The pooled IPD analyses on exercise-oncology trials so far have primarily been used to identify single treatment-covariate interactions, separately for each patient characteristic. ML models (eg, decision trees) facilitate analyzing combinations of covariables modifying intervention effects (ie, multiple levels of interactions). Additionally, large datasets allow division of data into a testing set and a validation set for ML models and training of AI. Recently, a conditional inference tree was applied to the IPD from the POLARIS database to examine which combination of patient and exercise characteristics were associated with dropout from the exercise program.^95^ The average dropout was 9.6%, but it varied between 5.1% and 19.8% depending on body mass index, intervention timing and supervision, and educational level.^66^ Applying ML/AI models to analyze which combinations of covariables predict treatment responses would further enhance precision exercise medicine.

Knowledge of the intervention moderators is not sufficient to guide decision making for precision exercise oncology, as this requires the ability to predict the treatment effect for each individual patient.^24^ To predict individual effects, ML models based on IPD of multiple RCTs can also be useful.^96^^,^^97^ For example, the IPD from the POLARIS database is currently being used to examine whether it can be predicted if individual patients would benefit more from psychosocial interventions or exercise interventions when aiming to reduce cancer-related fatigue. Other collaborators currently use the data to predict responses to exercise interventions. If with increasing numbers of studies over time, sufficient IPD is available, ML modeling and AI on pooled data opens exciting new avenues to enhance precision predictions.

## Conclusions

Pooled analyses on IPD of exercise oncology RCTs have been useful to inform precision exercise medicine for patients with cancer. Key findings from analyses to date highlight the importance of exercise supervision and specificity to maximize benefits and to consider a patient’s initial value of the outcome. Additionally, it has identified patient subgroups who are more likely to benefit from exercise and subgroups for whom more effective exercise programs need to be developed, such as for older and unfit patients with cancer. Yet, pooled IPD analyses have not been used to its full potential. Opportunities to further advance precision exercise medicine include pooling of data from understudied populations, data on clinical outcomes and biomarkers, and applying ML/AI methods for identifying combinations of covariables that modify intervention effects and predictions of individual treatment effects.

## Supplementary Material

pkaf078_Supplementary_Data

## Data Availability

No new data were generated or analyzed for this commentary.
