# Supplementary material for: Accelerating precision exercise medicine in cancer patients using pooled individual patient data: POLARIS experience
Source: JNCI Cancer Spectr. 2025 Aug 7;9(5):pkaf078. doi: 10.1093/jncics/pkaf078 (PMC12401491; doi:10.1093/jncics/pkaf078)
Supplement: pkaf078_Supplementary_Data [file pkaf078_supplementary_data.docx]

Table S1. Exercise oncology trials that are currently in the POLARIS database, categorized according to the phases of the cancer continuum using the Exercise Across the Postdiagnosis Cancer Continuum (EPiCC) framework

| **Author (year) *Acronym*** |  | **Country** | **N** | | **Age, mean (SD)** | | | **Gender,  (% female)** | **Cancer type**  **(% Distant metastases)** | | **Intervention** | | | **Exercise** |
| --- | --- | --- | --- | --- | --- | --- | --- | --- | --- | --- | --- | --- | --- | --- |
|  |  |  |  |  |  |  |  |  |  |  | **Delivery mode** | **Duration (weeks)** | | **FITT** |
| **PREHABILITATION EXERCISE STUDIES** | | | | | |  |  | | | |  |  |  |  |
| Banerjee et al. (2018) [1] |  | UK | 60 | | 72.1 (7.6) | | | 11.7 | Bladder  (0%) | | Supervised | 4 | | F: 2x/week  I: High  T: AE  T: 60 min |
| Barberan-Garcia et al. (2018) [2] |  | Spain | 96 | | 73.6 (8.0) | | | 20.8 | Unspecified  (0%) | | Supervised | Mean: 6 | | F: 1-3x/week  I: High  T: AE  T: 45 min |
| Bousquet-Dion et al. (2018) [3] |  | Canada | 63 | | 70.2 (10.6) | | | 27.0 | Colorectal  (0%) | | Supervised | Mean: 4.6 | | F: 3-4x/week  I: Moderate  T: AE + RE  T: 60 min |
| Carli et al. (2020) [4] |  | Canada | 110 | | 78.5 (7.3) | | | 52.7 | Colorectal  (0%) | | Supervised | 4 | | F: 4x/week  I: Moderate  T: AE + RE  T: 60 min |
| Dunne et al. (2016) [5] |  | UK | 37 | | 61.2 (9.8) | | | 29.7 | Colorectal liver metastasis  (100%) | | Supervised | 4 | | F: 3x/week  I: High  T: AE  T: 40 min |
| Jensen et al. (2015) [6] |  | Denmark | 107 | | 70.0 (9.5) | | | 15.9 | Bladder  (0%) | | Unsupervised | 2 | | F: 14x/week  I: Low-moderate  T: AE + RE  T: 30 min |
| Licker et al. (2017) [7] |  | Switzerland | 151 | | 63.5 (12.0) | | | 39.7 | Lung  (0%) | | Supervised | Median: 3 | | F: 2-3x/week  I: High  T: AE + RE  T: 35 min |
| Santa Mina et al. (2018) [8] |  | Canada | 87 | | 61.7 (7.4) | | | 0 | Prostate  (0%) | | Unsupervised | Median: 4.7 | | F: 3-4x/week  I: Moderate  T: AE + RE  T: 60 min |
| Sebio Garcia et al. (2017) [9] |  | Spain | 22 | | 70.1 (7.9) | | | 13.6 | Lung  (0%) | | Supervised | Median: 8.1 | | F: 3-5x/week  I: High  T: AE + RE  T: 60 min |
| **INTRAHABILITATION EXERCISE STUDIES** | | | | | |  |  | | | |  |  |  |  |
|  |  |  |  | |  | | |  |  | |  |  | |  |
| Bourke (2011) [10] |  | UK | 50 | | 72.0 (4.8) | | | 0 | Prostate cancer  (26%) | | Supervised | 12 | | F: First 6 weeks 2x/week supervised. Second 6 weeks 1x/week supervised  I: moderate-vigorous  T: AE + RE  T: AE: 30 min + RE |
| Mast (2025) [11] |  | NL | 31 | | 65.0 (8.0) | | | 16 | Esophageal  (0%) | | Supervised | 5 | | Exercise in hospital  F: 5x/week  I: moderate  T: AE  T: 30 min  Exercise with physiotherapist  F: 2x/week  I: moderate-vigorous  T: AE + RE  T: 60 min |
| Cadmus, (2009) [12]  *IMPACT* |  | USA | 50 | | 54.2 (9.6) | | | 100 | Breast  (0%) | | Unsupervised | 26 | | F: aim 5x/week I: moderate T: AE T: 30 min |
| Cormie (2015) [13] |  | AUS | 64 | | 67.9 (7.1) | | | 0 | Prostate  (0%) | | Supervised | 12 | | F: 2x/week I: moderate-vigorous T: AE + RE T: 60 min |
| Courneya (2003)* [14]  *CANHOPE* |  | CAN | 66 | | 59.8 (10.5) | | | 40.9 | Colorectal  (7.6%) | | Unsupervised | 16 | | F: 3-5x/week I: moderate T: AE T: 20–30 min |
| Courneya (2007) [15]  *START* |  | CAN | 242 | | 49.2 (9.3) | | | 100 | Breast  (0%) | | Supervised | Median: 17 | | F: 3x/week I: moderate-vigorous T: AE vs RE T: AE: 15–45 min |
| Courneya (2009) [16]  *HELP* |  | CAN | 54 | | 52.7 (15.3) | | | 27.8 | Hematological  (0%) | | Supervised | 12 | | F: 3x/week I: moderate-vigorous T: AE T: 15–45 min |
| Galvão (2010) [17] |  | AUS | 57 | | 69.8 (7.3) | | | 0 | Prostate  (0%) | | Supervised | 12 | | F: 2x/week I: moderate T: AE + RE T: 60 min |
| Galvão (2017) [18] *M3EP* |  | AUS | 57 | | 70.0 (8.4) | | | 0 | Prostate  (100%) | | Supervised | 12 | | F: 3x/week  I: moderate-vigorous  T: Aerobic, resistance, and flexibility  T: 60 min |
| Goedendorp (2010) [19] |  | NL | 144 | | 57.2 (10.5) | | | 63.2 | Mixed  (0%) | | Unsupervised | Mean: 31.7 | | F: towards 5d/week I: ? T: AE T: towards 60 min |
| Griffith (2009) [20] |  | USA | 126 | | 60.2 (10.6) | | | 38.9 | Mixed  (0%) | | Unsupervised | Mean: 12.8 | | F: 5x/week I: low-moderate T: AE T: 25-35 min |
| Hayes (2013)* [21]  *Exercise for Health* |  | AUS | 194 | | 52.4 (8.5) | | | 100 | Breast  (0%) | | Unsupervised | 35 | | F: aim: ⩾ 4x/week I: moderate T: AE + RE T: 20–45 min |
| Mutrie (2007) [22] |  | UK | 201 | | 51.6 (9.5) | | | 100 | Breast  (0%) | | Supervised | 12 | | F: 2x/week supervised  (+1x/week unsupervised) I: low-moderate T: AE + RE T: 45 min |
| Newton (2009) [23] |  | AUS | 154 | | 69.0 (9.0) | | | 0 | Prostate  (0%) | | Supervised | 24 | | F: 2x/week I: moderate-vigorous T: AE + RE vs RE + impact T: 60 min |
| Schmidt (2015) [24]  *BEATE* |  | GER | 88 | | 52.5 (10.0) | | | 100 | Breast  (0%) | | Supervised | 12 | | F: 2x/week I: moderate-vigorous T: RE T: 60 min |
| Steindorf (2014) [25]  *BEST* |  | GER | 141 | | 56.3 (8.9) | | | 100 | Breast  (0%) | | Supervised | 12 | | F: 2x/week I: moderate-vigorous T: RE T: 60 min |
| Taaffe (2017) [26] |  | AUS | 196 | | 67.5 (9.4) | | | 0 | Prostate  (0%) | | Supervised | 26 | | F: 3x/ week  I: moderate-vigorous  T: AE + RE + impact  T: 60 min |
| Travier (2015) [27]; van Vulpen (2016) [28] *PACT* |  | NL | 237 | | 50.7 (8.8) | | | 100 | Breast and Colon  (0%) | | Supervised | 18 | | F: 2x/week I: moderate-vigorous T: AE + RE T: 60 min |
| Van Waart (2015) [29]  PACES |  | NL | 253 | | 51.4 (9.5) | | | 95.7 | Breast and Colon  (0%) | | Unsupervised vs supervised | Mean: 15.9 | | F: supervised: 2x/week; unsupervised towards 5x/week I: supervised: moderate-vigorous Unsupervised: moderate T: supervised: RE + AE; unsupervised: AE T: supervised: 60 min; unsupervised: aim 30 min |
| Winters-Stone (2015) [30] |  | USA | 51 | | 70.1 (8.6) | | | 0 | Prostate  (21.6%) | | Supervised | 52 | | F: 2x/week supervised  (+1x/week unsupervised) I: moderate T: RE + impact T: 60 min |
| **REHABILITATION EXERCISE STUDIES** | | | |  | | | | |  |  | | |  |  |
| Arbane (2011) [31] |  | UK | 51 | | 64.0 (11.0) | | | 48.1 | Lung  (9.8%) | | Unsupervised | 12 | | F: ? I: moderate T: AE + RE T: ? |
| Courneya (2003) [32]  *REHAB* |  | CAN | 52 | | 58.6 (5.7) | | | 100 | Breast  (0%) | | Supervised | 15 | | F: 3x/week I: moderate-vigorous T: AE T: 15–35 min |
| Courneya (2003)* [14]  *CANHOPE* |  | CAN | 33 | | 61.6 (11.5) | | | 39.4 | Colorectal  (0%) | | Unsupervised | 16 | | F: 3-5x/week I: moderate T: AE T: 20–30 min |
| Courneya* (2009) [16]  *HELP* |  | CAN | 68 | | 53.5 (14.6) | | | 51.5 | Hematological  (0%) | | Supervised | 12 | | F: 3x/week I: moderate-vigorous T: AE T: 15–45 min |
| Daley (2007) [33] |  | UK | 108 | | 51.1 (8.6) | | | 100 | Breast  (0%) | | Supervised | 8 | | F: 3x/week I: moderate-vigorous T: AE T: 50 min |
| Duijts (2012) [34]  *EVA* |  | NL | 207 | | 47.8 (5.8) | | | 100 | Breast  (0%) | | Unsupervised | 12 | | F: 5x per 2 weeks I: vigorous T: AE T: 45–60 min^[⁎](https://www.sciencedirect.com/science/article/pii/S0305737216301359#tblfn1)^ |
| Galvão (2014) [35]  *RADAR-exercise* |  | AUS | 100 | | 71.7 (6.4) | | | 0 | Prostate  (0%) | | Supervised | 26 | | F: 2x/week I: moderate-vigorous T: RE + AE T: 60 min |
| Herrero (2006) [36] |  | Spain | 16 | | ? | | | 100 | Breast  (0%) | | Supervised | 8 | | F: 3x/week I: moderate-vigorous T: AE + RE T: 90 min |
| Irwin (2009) [37]  *YES* |  | USA | 75 | | 55.8 (8.7) | | | 100 | Breast  (0%) | | Supervised | 26 | | F: 3x/week supervised  (+ 2x/week unsupervised) I: moderate T: AE (walking) T: 15–30 min |
| Kampshoff (2015) [38]  *REACT* |  | NL | 277 | | 53.5 (11.0) | | | 80.1 | Mixed  (0%) | | Supervised | 12 | | F: 2x/week I: moderate vs vigorous T: AE + RE T: 60 min |
| Korstjens (2008) [39]  *OncoRev* |  | NL | 133 | | 50.6 (10.2) | | | 85 | Mixed  (18%) | | Supervised | 12 | | F: 2x/week I: AE: moderate-vigorous, RE: low-moderate T: AE + RE T: 120 min |
| Mehnert (2011) [40] |  | GER | 58 | | 51.9 (8.5) | | | 100 | Breast  (0%) | | Supervised | 10 | | F: 2x/week I: moderate T:AE + gymnastics + movement games + relaxation T: 90 min |
| Northey (2018) [41] |  | AUS | 17 | | 62.9 (7.9) | | | 100 | Breast cancer  (0%) | | Supervised | 12 | | F: 3x/week  I: Moderate intervention:  Moderate  HIIT intervention: high  T: AE  T: 20-30 min |
| Ohira (2006) [42]  *WTBS* |  | USA | 86 | | 52.7 (8.3) | | | 100 | Breast  (0%) | | Supervised | 26 (13 supervised) | | F: 2x/week I: ? T: RE T: ? |
| Persoon, (2010) [43] *EXIST* |  | NL | 109 | | 52.4 (11.2) | | | 36.7 | Hematological  (0%) | | Supervised | 18 | | F: 2x/week I: moderate-vigorous T: AE + RE T: 60 min |
| Speck (2010) [44]  *PAL* |  | USA | 295 | | 56.0 (8.8) | | | 100 | Breast  (0%) | | Supervised | 52 (13 supervised | | F: 2x/week I: ? T: RE T: 90 min |
| Thorsen (2005) [45] |  | NOR | 139 | | 39.4 (8.3) | | | 67.1 | Mixed  (21%) | | Unsupervised | 14 | | F: 2x/week or more I: moderate-vigorous T: AE + RE T: 30 min or more |
| Van Vulpen (2021) [46] *PERFECT* |  | NL | 120 | | 63.7 (8.1) | | | 13.3 | Esophageal  (0%) | | Supervised | 12 | | F: 2x/week  I: moderate-vigorous  T: AE + RE  T: 60 min |
| Winters-Stone (2012) [47] |  | USA | 106 | | 62.2 (6.7) | | | 100 | Breast  (0%) | | Supervised | 52 | | F: 2x/week supervised  (+ 1x/week unsupervised) I: moderate-vigorous T: RE + impact T: 60 min |
| Winters-Stone (2013) [48] |  | USA | 71 | | 46.4 (4.9) | | | 100 | Breast  (0%) | | Supervised | 52 | | F: 2x/week supervised  (+ 1x/week unsupervised) I: moderate T: RE + impact T: 60 min |
| Wiskemann* (2011) [49] |  | GER | 80 | | 48.4 (14.4) | | | 31.3 | Hematological  (0%) | | Supervised | Median exercise: 16.4 Control: 15.7 | | F: 5x/week I: moderate-vigorous T: AE + RE T: AE: 20–40 min |
| **HEALTH PROMOTION AND DISEASE PREVENTION EXERCISE** | | | | | | | | | | |  |  |  |  |
| Lahart (2016) [50] |  | UK | 80 | | 53.6 (9.4) | | | 100 | Breast  (0%) | | Unsupervised | 26 | | F: First three months 3-5x/week  Last three months 5-7x/week  I: moderate  T: ?  T: 30 min |
| McNeil (2022) [51]  *BC-PAL* |  | Canada | 45 | | 58.5 (9.3) | | | 100 | Breast  (0%) | | Unsupervised | 12 | | F: ?  I: Lower intensity intervention: low-moderate  Higher intensity intervention: high  T: AE  T: ? |
| Short (2015) [52]  *MM4L* |  | AUS | 330 | | 55.9 (8.3) | | | 100 | Breast  (1.8%)) | | Unsupervised | 16 | | F: AE: 5x/week; RE: 1-3x/week I: moderate T: AE + RE T: AE: 30 min |

ADT = androgen deprivation therapy; AE = Aerobic exercise training; allo-HSCT = allogeneic hematopoietic stem cell transplantation; CT = chemotherapy; FITT=Frequency, Intensity, Type, Time; RE = Resistance exercise training; RT = radiotherapy; SCT = stem cell transplantation.

*Study that is located in multiple places across the cancer continuum according EPiCC framework [53].

**References**

1. Banerjee, S., et al., *Vigorous intensity aerobic interval exercise in bladder cancer patients prior to radical cystectomy: a feasibility randomised controlled trial.* Supportive Care in Cancer, 2018. **26**: p. 1515-1523.

2. Barberan-Garcia, A., et al., *Personalised prehabilitation in high-risk patients undergoing elective major abdominal surgery: a randomized blinded controlled trial*. 2018, LWW.

3. Bousquet-Dion, G., et al., *Evaluation of supervised multimodal prehabilitation programme in cancer patients undergoing colorectal resection: a randomized control trial.* Acta Oncologica, 2018. **57**(6): p. 849-859.

4. Carli, F., et al., *Effect of multimodal prehabilitation vs postoperative rehabilitation on 30-day postoperative complications for frail patients undergoing resection of colorectal cancer: a randomized clinical trial.* JAMA surgery, 2020. **155**(3): p. 233-242.

5. Dunne, D., et al., *Randomized clinical trial of prehabilitation before planned liver resection.* Journal of British Surgery, 2016. **103**(5): p. 504-512.

6. Jensen, B.T., et al., *Efficacy of a multiprofessional rehabilitation programme in radical cystectomy pathways: a prospective randomized controlled trial.* Scandinavian journal of urology, 2015. **49**(2): p. 133-141.

7. Licker, M., et al., *Short-term preoperative high-intensity interval training in patients awaiting lung cancer surgery: a randomized controlled trial.* Journal of thoracic oncology, 2017. **12**(2): p. 323-333.

8. Santa Mina, D., et al., *Prehabilitation for radical prostatectomy: a multicentre randomized controlled trial.* Surgical oncology, 2018. **27**(2): p. 289-298.

9. Sebio García, R., et al., *Preoperative exercise training prevents functional decline after lung resection surgery: a randomized, single-blind controlled trial.* Clinical rehabilitation, 2017. **31**(8): p. 1057-1067.

10. Bourke, L., et al., *Lifestyle intervention in men with advanced prostate cancer receiving androgen suppression therapy: a feasibility study.* Cancer Epidemiology, Biomarkers & Prevention, 2011. **20**(4): p. 647-657.

11. Ubink, A., et al., *Exploring the effects of exercise on immune cell function and tumour infiltration in patients with breast cancer receiving neoadjuvant chemotherapy - a feasibility trial.* Brain Behav Immun Health, 2025. **46**: p. 101021.

12. Cadmus, L.A., et al., *Exercise and quality of life during and after treatment for breast cancer: results of two randomized controlled trials.* Psycho‐Oncology: Journal of the Psychological, Social and Behavioral Dimensions of Cancer, 2009. **18**(4): p. 343-352.

13. Cormie, P., et al., *Can supervised exercise prevent treatment toxicity in patients with prostate cancer initiating androgen‐deprivation therapy: a randomised controlled trial.* BJU international, 2015. **115**(2): p. 256-266.

14. Courneya, K., et al., *A randomized trial of exercise and quality of life in colorectal cancer survivors.* European journal of cancer care, 2003. **12**(4): p. 347-357.

15. Courneya, K.S., et al., *Effects of aerobic and resistance exercise in breast cancer patients receiving adjuvant chemotherapy: a multicenter randomized controlled trial.* J Clin Oncol, 2007. **25**(28): p. 4396-4404.

16. Courneya, K.S., et al., *Randomized controlled trial of the effects of aerobic exercise on physical functioning and quality of life in lymphoma patients.* J Clin Oncol, 2009. **27**(27): p. 4605-4612.

17. Galvao, D.A., et al., *Combined resistance and aerobic exercise program reverses muscle loss in men undergoing androgen suppression therapy for prostate cancer without bone metastases: a randomized controlled trial.* Journal of clinical oncology, 2010. **28**(2): p. 340-347.

18. Galvao, D.A., et al., *Exercise preserves physical function in prostate cancer patients with bone metastases.* Medicine and science in sports and exercise, 2017. **50**(3): p. 393.

19. Goedendorp, M.M., et al., *Is increasing physical activity necessary to diminish fatigue during cancer treatment? Comparing cognitive behavior therapy and a brief nursing intervention with usual care in a multicenter randomized controlled trial.* The oncologist, 2010. **15**(10): p. 1122-1132.

20. Griffith, K., et al., *Impact of a walking intervention on cardiorespiratory fitness, self‐reported physical function, and pain in patients undergoing treatment for solid tumors.* Cancer, 2009. **115**(20): p. 4874-4884.

21. Hayes, S.C., et al., *Exercise for health: a randomized, controlled trial evaluating the impact of a pragmatic, translational exercise intervention on the quality of life, function and treatment-related side effects following breast cancer.* Breast cancer research and treatment, 2013. **137**: p. 175-186.

22. Mutrie, N., et al., *Benefits of supervised group exercise programme for women being treated for early stage breast cancer: pragmatic randomised controlled trial.* Bmj, 2007. **334**(7592): p. 517.

23. Newton, R.U., et al., *A phase III clinical trial of exercise modalities on treatment side-effects in men receiving therapy for prostate cancer.* BMC cancer, 2009. **9**(1): p. 1-8.

24. Schmidt, M.E., et al., *Effects of resistance exercise on fatigue and quality of life in breast cancer patients undergoing adjuvant chemotherapy: a randomized controlled trial.* International journal of cancer, 2015. **137**(2): p. 471-480.

25. Steindorf, K., et al., *Randomized, controlled trial of resistance training in breast cancer patients receiving adjuvant radiotherapy: results on cancer-related fatigue and quality of life.* Annals of oncology, 2014. **25**(11): p. 2237-2243.

26. Taaffe, D.R., et al., *Effects of different exercise modalities on fatigue in prostate cancer patients undergoing androgen deprivation therapy: a year-long randomised controlled trial.* European Urology, 2017. **72**(2): p. 293-299.

27. Travier, N., et al., *Effects of an 18-week exercise programme started early during breast cancer treatment: a randomised controlled trial.* BMC medicine, 2015. **13**(1): p. 1-11.

28. Van Vulpen, J.K., et al., *Effects of an exercise program in colon cancer patients undergoing chemotherapy.* Med Sci Sports Exerc, 2016. **48**(5): p. 767-775.

29. Van Waart, H., et al., *Effect of low-intensity physical activity and moderate-to high-intensity physical exercise during adjuvant chemotherapy on physical fitness, fatigue, and chemotherapy completion rates: results of the PACES randomized clinical trial.* J Clin Oncol, 2015. **33**(17): p. 1918-1927.

30. Winters-Stone, K.M., et al., *Resistance training reduces disability in prostate cancer survivors on androgen deprivation therapy: evidence from a randomized controlled trial.* Archives of physical medicine and rehabilitation, 2015. **96**(1): p. 7-14.

31. Arbane, G., et al., *Evaluation of an early exercise intervention after thoracotomy for non-small cell lung cancer (NSCLC), effects on quality of life, muscle strength and exercise tolerance: randomised controlled trial.* Lung cancer, 2011. **71**(2): p. 229-234.

32. Courneya, K.S., et al., *Randomized controlled trial of exercise training in postmenopausal breast cancer survivors: cardiopulmonary and quality of life outcomes.* Journal of clinical oncology, 2003. **21**(9): p. 1660-1668.

33. Daley, A.J., et al., *Randomized trial of exercise therapy in women treated for breast cancer.* Journal of clinical oncology, 2007. **25**(13): p. 1713-1721.

34. Duijts, S., et al., *Efficacy of cognitive behavioral therapy and physical exercise in alleviating treatment-induced menopausal symptoms in patients with breast cancer: results of a randomized, controlled, multicenter trial.* J Clin Oncol, 2012. **30**(33): p. 4124-4133.

35. Galvao, D.A., et al., *A multicentre year-long randomised controlled trial of exercise training targeting physical functioning in men with prostate cancer previously treated with androgen suppression and radiation from TROG 03.04 RADAR.* European urology, 2014. **65**(5): p. 856-864.

36. Herrero, F., et al., *Combined aerobic and resistance training in breast cancer survivors: A randomized, controlled pilot trial.* International journal of sports medicine, 2006. **27**(07): p. 573-580.

37. Irwin, M.L., et al., *Randomized controlled trial of aerobic exercise on insulin and insulin-like growth factors in breast cancer survivors: the Yale Exercise and Survivorship study.* Cancer Epidemiology Biomarkers & Prevention, 2009. **18**(1): p. 306-313.

38. Kampshoff, C.S., et al., *Randomized controlled trial of the effects of high intensity and low-to-moderate intensity exercise on physical fitness and fatigue in cancer survivors: results of the Resistance and Endurance exercise After ChemoTherapy (REACT) study.* BMC Med, 2015. **13**: p. 275.

39. Korstjens, I., et al., *Quality of life after self-management cancer rehabilitation: a randomized controlled trial comparing physical and cognitive-behavioral training versus physical training.* Psychosomatic Medicine, 2008. **70**(4): p. 422-429.

40. Mehnert, A., et al., *Effects of a physical exercise rehabilitation group program on anxiety, depression, body image, and health-related quality of life among breast cancer patients.* Oncology Research and Treatment, 2011. **34**(5): p. 248-253.

41. Northey, J.M., et al., *Cognition in breast cancer survivors: a pilot study of interval and continuous exercise.* Journal of science and medicine in sport, 2019. **22**(5): p. 580-585.

42. Ohira, T., et al., *Effects of weight training on quality of life in recent breast cancer survivors: the Weight Training for Breast Cancer Survivors (WTBS) study.* Cancer: Interdisciplinary International Journal of the American Cancer Society, 2006. **106**(9): p. 2076-2083.

43. Persoon, S., et al., *Design of the EXercise Intervention after Stem cell Transplantation (EXIST) study: a randomized controlled trial to evaluate the effectiveness and cost-effectiveness of an individualized high intensity physical exercise program on fitness and fatigue in patients with multiple myeloma or (non-) Hodgkin's lymphoma treated with high dose chemotherapy and autologous stem cell transplantation.* BMC cancer, 2010. **10**: p. 1-9.

44. Speck, R.M., et al., *Changes in the Body Image and Relationship Scale following a one-year strength training trial for breast cancer survivors with or at risk for lymphedema.* Breast cancer research and treatment, 2010. **121**: p. 421-430.

45. Thorsen, L., et al., *Effectiveness of physical activity on cardiorespiratory fitness and health-related quality of life in young and middle-aged cancer patients shortly after chemotherapy.* Journal of Clinical Oncology, 2005. **23**(10): p. 2378-2388.

46. van Vulpen, J., et al., *Supervised exercise after oesophageal cancer surgery: the PERFECT multicentre randomized clinical trial.* British Journal of Surgery, 2021. **108**(7): p. 786-796.

47. Winters-Stone, K.M., et al., *The effect of resistance training on muscle strength and physical function in older, postmenopausal breast cancer survivors: a randomized controlled trial.* Journal of Cancer Survivorship, 2012. **6**: p. 189-199.

48. Winters-Stone, K., et al., *Impact+ resistance training improves bone health and body composition in prematurely menopausal breast cancer survivors: a randomized controlled trial.* Osteoporosis international, 2013. **24**: p. 1637-1646.

49. Wiskemann, J., et al., *Effects of a partly self-administered exercise program before, during, and after allogeneic stem cell transplantation.* Blood, The Journal of the American Society of Hematology, 2011. **117**(9): p. 2604-2613.

50. Lahart, I.M., et al., *Randomised controlled trial of a home-based physical activity intervention in breast cancer survivors.* BMC cancer, 2016. **16**: p. 1-14.

51. McNeil, J., et al., *Adherence to a lower versus higher intensity physical activity intervention in the Breast Cancer & Physical Activity Level (BC-PAL) Trial.* Journal of Cancer Survivorship, 2022: p. 1-13.

52. Short, C.E., et al., *Main outcomes of the Move More for Life Trial: A randomised controlled trial examining the effects of tailored‐print and targeted‐print materials for promoting physical activity among post‐treatment breast cancer survivors.* Psycho‐oncology, 2015. **24**(7): p. 771-778.

53. Courneya, K.S., et al., *An integrated framework for the study of exercise across the postdiagnosis cancer continuum.* Frontiers in Oncology, 2024. **14**: p. 1432899.
